# Complete Mitochondrial Genome and Phylogenetic Analysis of the Blue Whistling Thrush (*Myophonus caeruleus*)

**DOI:** 10.3390/genes15070830

**Published:** 2024-06-24

**Authors:** Zhenfeng Yuan, Peng Liu, Xi Lu, Dong Zhu, Jun Liu, Qiang Guo, Wenping Zhang, Yubao Duan

**Affiliations:** 1 Key Laboratory for Forest Resources Conservation and Utilization in the Southwest Mountains of China, Ministry of Education, Southwest Forestry University, Kunming 650224, China; yzf25802023@163.com (Z.Y.); 19373300545@163.com (P.L.); z2607857825@gmail.com (D.Z.); guoq9382@163.com (Q.G.); 2 Key Laboratory for Conserving Wildlife with Small Populations in Yunnan, Southwest Forestry University, Kunming 650224, China; lx95212024@163.com (X.L.); liu1093799514@163.com (J.L.); 3 Key Laboratory of Forest Disaster Warning and Control in Yunnan Province, Kunming 650224, China; 4 College of Forestry, Southwest Forestry University, Kunming 650224, China; 5 Key Laboratory of Monitoring Biological Diversity in Minshan Mountain of National Park of Giant Pandas, College of Life Science & Biotechnology, Mianyang Normal University, Mianyang 621000, China

**Keywords:** Muscicapidae, mitogenomic phylogeny, mitogenome, *Myophonus caeruleus*

## Abstract

The blue whistling thrush (*Myophonus caeruleus*) is a bird belonging to the order Passeriformes and family Muscicapidae. *M. caeruleus* is widely distributed in China, Pakistan, India, and Myanmar and is a resident bird in the southern part of the Yangtze River in China and summer migratory bird in the northern part of the Yangtze River. At present, there are some controversies about the classification of *M. caeruleus.* We use complete mitochondrial genomes to provide insights into the phylogenetic position of *M. caeruleus* and its relationships among Muscicapidae. The mitochondrial genome (GenBank: MN564936) is 16,815 bp long and contains 13 protein-coding genes (PCGs), 2 rRNA genes, 22 tRNA genes, and a non-coding control region (D-loop). The thirteen PCGs started with GTG and ATG and ended with five types of stop codons. The nucleotide composition of T was 23.71%, that of C was 31.45%, that of A was 30.06%, and that of G was 14.78%. The secondary structures of 22 tRNAs were predicted, all of which could form typical cloverleaf structures. There were 24 mismatches, mainly G–U mismatches. Through phylogenetic tree reconstruction, it was found that *Saxicola*, *Monticola*, *Oenanthe*, and *Phoenicurus* were clustered into one clade, together with the sister group of *Myophonus*.

## 1. Introduction

The mitochondrial genome is the genetic material within the mitochondria, most of which follows strict maternal inheritance, is highly conserved, and is almost unaffected by gene recombination [1]. Avian mitochondrial genomes are easy to extract and amplify, making them ideal markers for molecular-level evolutionary analysis [2,3]. The length of the avian mitochondrial DNA genome is about 16.3 kb to 20 kb and consists of one heavy chain (H) and one light chain (L) forming a closed circular double-stranded molecule [4]. It is usually composed of 37 genes, including 13 protein-coding genes (PCGs), 22 transfer RNA (tRNAs), 2 ribosomes RNA (rRNAs), and a large non-coding D-lLoop region [5,6]; in addition, there are many species that retain the pseudo-D-loop region, such as *Sinosuthora conspicillata* and *Falco rusticolus* [7,8]. The characteristics of mitochondrial genes in birds are due to a rearrangement near the control region that differs from the gene order found in most vertebrates. In addition, most mitochondrial DNA mutations are point mutations with few insertions or deletions [1]. At the same time, compared with a single gene that can only provide limited information, the complete sequence of the mitochondrial genome contains more abundant and accurate information. Because of these advantages, avian mitochondrial genomes have been widely used in phylogenetic reconstruction and interspecific genetic diversity analysis [9]. 

**Figure 1:**
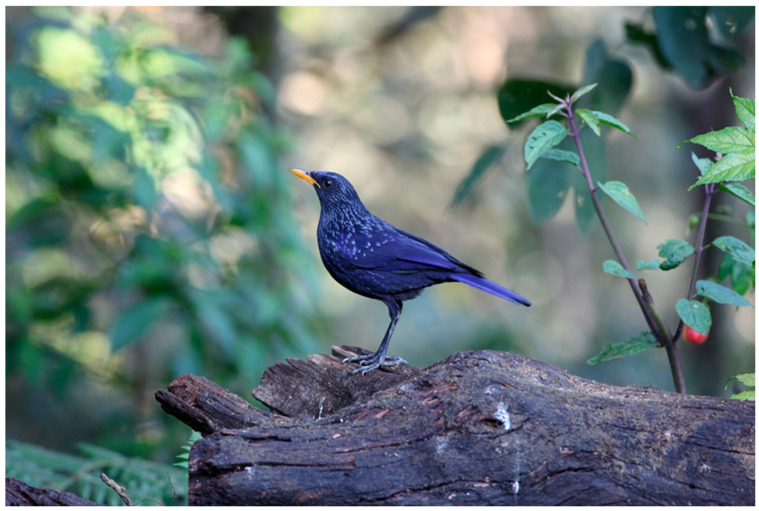
A picture of *Myophonus caeruleus*. The photo was taken by Jun Liu on 15 January 2020 at Baihualing of Gaoligong Mountain in Baoshan City, Yunnan province, China.

The blue whistling thrush (*Myophonus caeruleus*) is a bird belonging to the order Passeriformes and family Muscicapidae, and it occupies mountains and stream sides [10]. *M*. *caeruleus* has a dark bluish-purple body, with pale bluish-purple spots and markings all over the body except for the wings and tail and a yellow beak (Figure 1). There are nine species of *Myophonus* in the world, the Taiwan whistling thrush (*M. insularis*), blue whistling thrush (*M. caeruleus*), Ceylon whistling thrush (*M. lighi*), shiny whistling thrush (*M. melanurus*), Sunda whistling thrush (*M. glaucinus*), Bornean whistling thrush (*M. borneensis*), brown-winged whistling thrush (*M. castaneus*), Malayan whistling thrush (*M. robinsoni*) and Malabar whistling thrush (*M. horsfieldii*) [10]. Among them, the first two species are distributed in China and the last seven species are found in other countries. *M. insularis* is distributed only in the Taiwan Province. *M. caeruleus* is found in Central Asia, East Asia, and Southeast Asia. It is widely distributed in China, including Xizang and central and southern provinces of China.

At present, phylogenetic relationships of Muscicapidae have been studied using mtDNA and nDNA [11], but the complete mitochondrial genome sequence for *M. caeruleus* is lacking. To better understand the mitochondrial genome characteristics and phylogenetic relationships of *M. caeruleus*, we sequenced the *M. caeruleus* mitochondrial genome based on next-generation sequencing and reconstructed the *M. caeruleus* phylogenetic relationship in combination with published data.

## 2. Materials and Methods

### 2.1. Samples and DNA Extraction

The sample of *M. caeruleus* was an individual who died of natural causes in the wild in Zhaotong City of Yunnan Province, China. All tissues used in this study were preserved in absolute ethanol and stored at −20 °C until DNA extraction. Using the TIANamp Genomic DNA Kit (DP304, TIANGEN, Beijing, China), DNA was extracted from the muscle according to the instructions. The DNA integrity was determined by agarose gel electrophoresis at 1% concentration, and the DNA concentration and purity were measured on a NanoDrop 2000 spectrophotometer (NanoDrop Technologies, Wilmington, DE, USA).

### 2.2. Genome Sequencing, Assembly, and Annotation

The DNA extract was sent to Shanghai Paisenor Biotechnology Co., Ltd. (Shanghai, China), to construct a library using the whole-genome shotgun (WGS) strategy and utilize next-generation sequencing (NGS) technology. These libraries featured paired-end (PE) sequencing based on the Illumina MiSeq sequencing platform. The mitogenome sequence is stored in GenBank database (https://www.ncbi.nlm.nih.gov/genbank/, accessed on 19 June 2024) with the accession number MN564936. High-quality second-generation sequencing data were assembled from scratch using A5-miseq v20150522 [12] and SPAdesv3.9.0 [13]. The results were corrected using Pilon v1.18 [14] software to obtain the final mitochondrial sequence. The tRNA was validated by the MITOS WebServer [15] (http://mitos.bioinf.uni-leipzig.de, accessed on 19 June 2024); tRNAscan-SE 2.0 [16] adjusted the default setting to the vertebrate mitochondrial genetic code, and tRNAscan-SE 2.0 was used to predict the secondary structure of the tRNA. The open reading frame (ORF) finder [17] on NCBI was used to identify the protein-coding region, set the vertebrate mitochondrial genetic code, and translate it into specific proteins using GenBank. The base composition was calculated and the relative synonymous codon usage (RSCU) was analyzed using MEGA 7.0 [18]. The combined skewness was calculated using the formula “AT-skew = (A − T)/(A + T)” and “GC-skew = (G − C)/(G + C)”. The CGView Server was used to map the genome (http://stothard.afns.ualberta.ca/cgview_server/index.html, accessed on 19 June 2024) [19].

### 2.3. The Phylogenetic Position of M. caeruleus

In addition to the species targeted in this study, 16 complete mitochondrial genomes were downloaded from the GenBank database. The phylogenetic location of *M. caeruleus* in Muscicapidae was determined through a comparison of 17 complete mitochondrial genomes (Table 1). The outgroup selected was *Paradoxornis heudei* (NC_046943), which belongs to family Paradoxornithidae. In addition, HQ896033 was published as *Cyanoptila cyanomelana* and is still listed on GenBank as that species but is actually a misidentified *Cyornis hainanus* or *C. rubeculoides*. We used these sequences with the correct species labels. MEGA 7.0 [18] was used to compare 20 sequences and remove the missing base parts. Bayesian inference (BI) was used for the phylogenetic analysis. The Bayesian information criterion (BIC) in jModelTest v.0.1.1 was used to determine the optimal nucleotide replacement model as GTR + G + I [20]. Using MrBayes [21], we constructed a Bayesian inference (BI) phylogenetic tree based on 13 PCGs, respectively. Four Markov chains in the BI phylogenetic tree ran simultaneously, totaling 400,000 generations. Samples were collected every 100 generations, and the first 25% was discarded as burnin [22]. The phylogenetic trees were visualized with FigTree v.1.2.2.

## 3. Results

### 3.1. Organization and Structure of M. caeruleus Mitochondrial Genome

**Figure 2:**
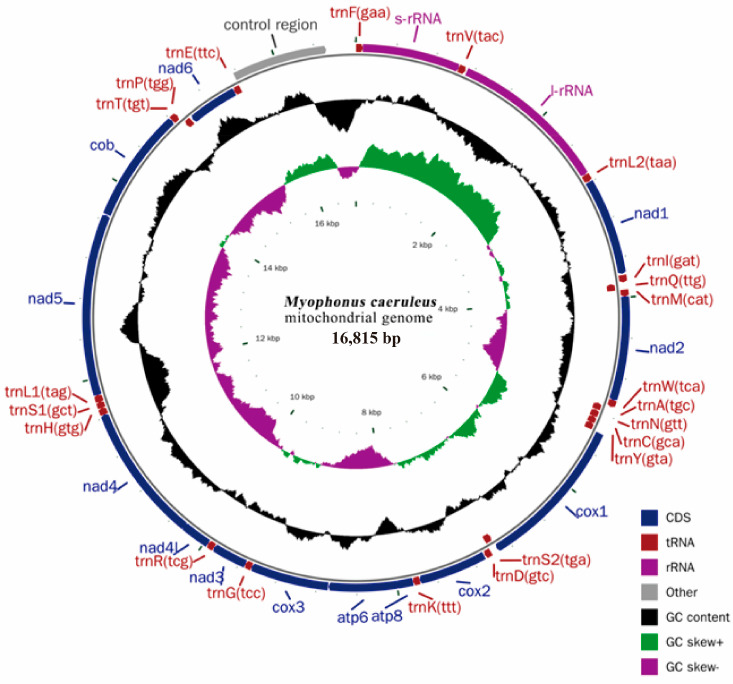
Complete mitochondrial gene map of *M. caeruleus*.

The mitochondrial genome of *M. caeruleus* (GenBank: MN564936) is 16,815 bp in length, of which the mitochondrial genome coding region is 15,568 bp in length and contained 37 genes, including 13 protein-coding genes, 2 rRNA genes, 22 tRNA genes, and a non-coding region (Figure 2). They accounted for 62.30%, 15.50%, 8.94%, and 5.75% of the mtDNA’s total length, respectively. There are nine genes in the L chain, including eight tRNA genes and one protein-coding gene (ND6), and the rest of the protein-coding genes are encoded through the H-chain.

The arrangement of genes in the mtDNA of *M. caeruleus* is relatively tight, and there are 32 bp (nine places) gene overlaps among 37 genes. There are a total of 22 gene intervals, with a length of 402 bp, accounting for only 2.43% of the total length of mitochondrial genes. The interval length ranged from 1 to 296 bp. The genes are closely arranged in seven places without overlapping or spacing. The mitochondrial genome analysis of *M. caeruleus* is shown in Table 2. MEGA 7.0 software was used to calculate the base composition of the complete mitochondrial genome as A = 30.06%, T = 23.71%, C = 31.45%, and G = 14.78%. Among them, the content of A + T base (53.77%) was slightly higher than that of G + C base (46.23%), and the skew of AT (AT-skew) was 0.118, while that of GC (GC-skew) was −0.36 (Table 3).

### 3.2. rRNA and tRNA Genes

Both the s-rRNA and l-rRNA genes of *M. caeruleus* are on the H strand. s-rRNA is 980 bp long and is located between trnF and trnV. l-rRNA is 1579 bp long and lies between trnV and trnL2. The A + T content of the two rRNA is s-rRNA = 51.94%, and the AT-skew is 0.202. The l-rRNA = 55.67%, and the AT-skew is 0.24.

**Figure 3:**
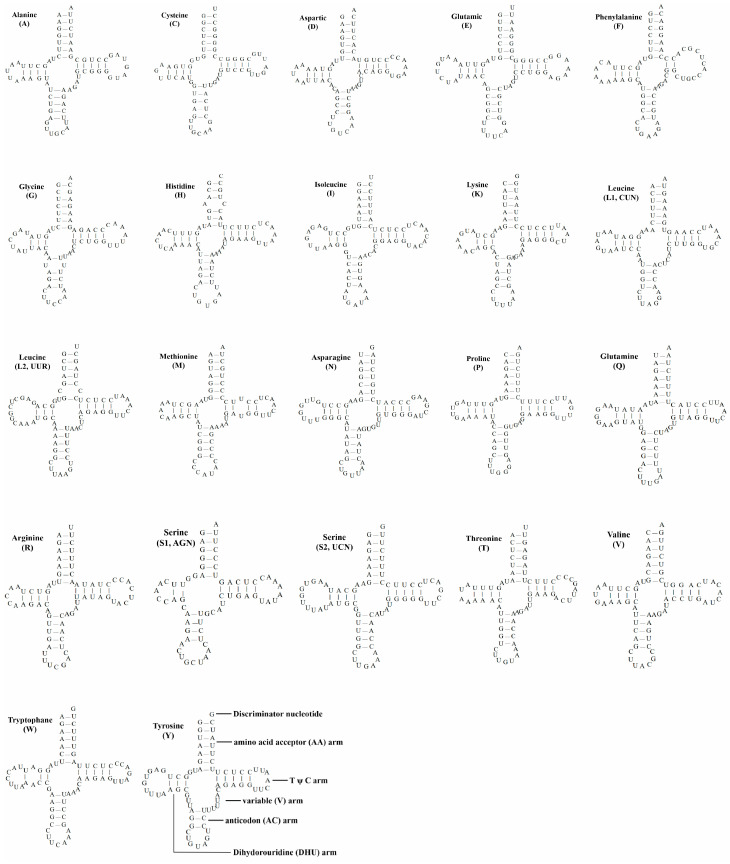
The twenty-two tRNAs as the secondary structure of *M. caeruleus*.

There are 22 tRNA genes distributed between protein-coding genes and rRNA genes in the mitochondria of *M. caeruleus*. The total length of tRNA genes is 1546 bp, and the length of single genes ranged from 66 (trnS1) to 75 bp (trnL2 and trnS2). The construction of the predicted secondary structure of tRNA is found to be able to form a clover structure (Figure 3). There are 24 mismatches in 22 tRNAs, including 6 pairs on the DHU arm, 3 pairs on anticodon arm, 7 pairs on TΨC arm, and 8 pairs on amino acid arm, all of which are GU mismatches.

### 3.3. Protein-Coding Genes Composition

**Figure 4:**
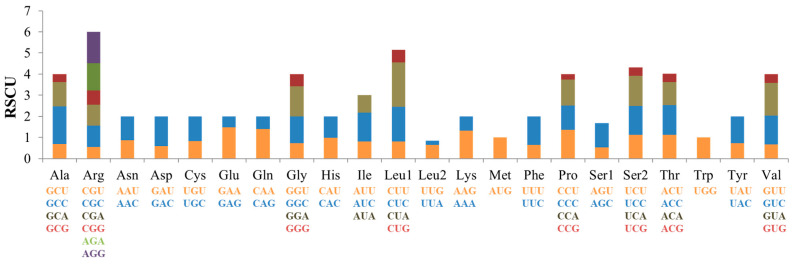
The relative codon usage frequency of the 13 PCGs.

The total length of the 13 protein-coding genes is 10,246 bp, accounting for 60.93% of the total mitochondrial genome length. Among the 13 PCGs, only ND6 is encoded by the L strand, and the other 12 genes are encoded by the H chain. The ND5 gene had the longest length (1818 bp). The shortest gene is Atp8 with a length of 168 bp. In this study, the initial codon of *M. caeruleus* is usually ATG, but the start codon of COI was GTG. The stop codon is dominated by TAA. Among them, ND2, COII, Atp8, Atp6, ND3, and ND4l have TAA as the termination codons; COI has AGG as the termination codons; ND5 has AGA as the termination codons; ND6 has TAG as the termination codons; and ND1, COIII, and ND4 have incomplete termination codons. They are TA- and T-, respectively (Table 2). The highest RSCU of PCGs is CUA (2.35). The lowest used codon is GCG with a frequency of 0.25 (Table 4, Figure 4).

### 3.4. Non-Coding Sequencin

*M. caeruleus* has one control region. The total length of the mtDNA control region is 950 bp, located between trnE and trnF. The base combination in the D-loop region is: A: 22.32%; T: 30.63%; G: 17.68%; and C: 29.37%. The content of A + T (52.95%) is slightly higher than that of G + C (47.05%), indicating bias.

### 3.5. Phylogenetic Analysis

**Figure 5:**
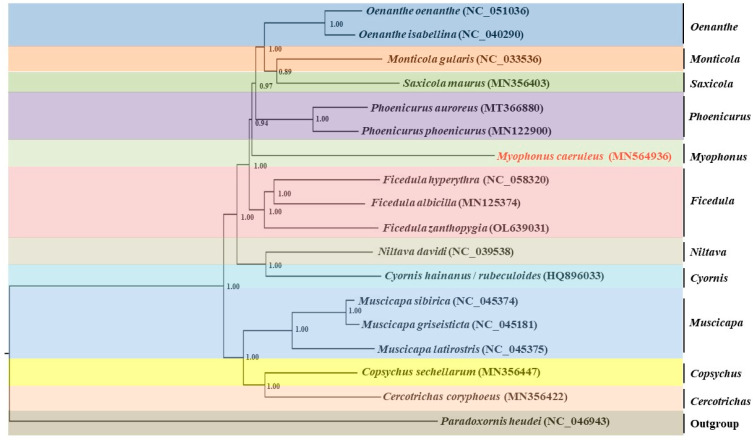
Phylogeny of mitochondrial genome sequence of *M. caeruleus*. Topology of Bayesian inference (BI) analysis inferred from the protein-coding genes.

In order to construct the phylogenetic tree, the complete mitochondrial genomes of 16 species of passerine birds from 12 genera in two families were searched and downloaded from the NCBI. This included one *Myophonus*, three *Ficedula*, one *Niltava*, two *Muscicapa*, one *Copsychus*, one *Cercotrichas*, one *Saxicola*, one *Monticola*, two *Oenanthe*, one *Cyornis*, and two *Phoenicurus* genera. *C. heudei* is an outgroup, and these sequences are constructed based on 13 PCGs. A BI phylogeny reconstructed using RAxML-GUI is shown in Figure 5.

Our results show that in the phylogenetic trees, all species are divided at the genus level into two large clades, with *Muscicapa*, *Copsychus*, and *Cercotrichas* clustered into one large clade and *Oenanthe*, *Monticola*, *Saxicola*, *Phoenicurus*, *Myophonus*, *Ficedula*, *Niltava*, and *Cyornis* clustered into another large clade. In the macroclades, *Saxicola* and *Monticola* are grouped together and were sister to *Oenanthe*. Some of these three genera are sister to *Phoenicurus* (BI bootstrap support 0.97). *M. caeruleus* is grouped with *Saxicola*, *Monticola*, *Oenanthe*, and *Phoenicurus* (BI bootstrap support 0.94). Among them, these four genera are grouped into a single branch and were sister to *Myophonus*. Furthermore, *Ficedula* is the sister clade of (*Niltava* + *Cyornis*).

## 4. Discussion

### 4.1. Mitogenome Characteristics

The Muscicapidae is the largest group of Passeriformes, with 312 species and 709 subspecies in 49 genera. Like other birds, the mitochondrial genome of *M. caeruleus* is covalent circular double-stranded, consisting of 37 genes, 13 PCGs, 2 rRNA genes (rrnL and rrnS), 22 tRNA genes, and 1 non-coding control region (CR) [2,5]. According to the literature, the mtDNA length of birds is generally between 15 and 20 kb. The mitochondrial genome of *M. caeruleus* in this paper is within this range.

Similar to other birds [32], gene overlap and gene spacing exist in the mitochondrial genome of *M. caeruleus*. The gene overlap was 1–10 bp, and the gene interval was 1–17 bp. The gene distribution of this species was the same as that of most vertebrates [33]. Except for NAD6 and eight trnas (trnQ, trnA, trnN, trnC, trnY, trnS2, trnP, and trnE), all genes were evenly distributed on the H strand. The mitochondrial A + T content of the whole genome was 53.77%, which was consistent with the typical base bias of vertebrates described by Huang L. [34]. The average length of tRNA was 70 bp, the longest was trnL and trnS2 (75 bp), and the shortest was trnS1 (66 bp). All tRNAs can be folded into a standard clover model. The two rRNAs were rrnS and rrnL, with lengths of 980 bp and 1579 bp, respectively, and the two rRNAs were located between trnF and trnL2 and were separated by trnV, as in most vertebrates. The start codon of PCGs was usually ATC, but it was GTG in CO I, consistent with previous findings [35]. Four complete terminal codons have been identified, namely TAA (NAD2, COII, Atp8, Atp6, NAD3, NAD4l, cob), AGG (COI), AGA (NAD5), and TAG (NDA6). For NAD1, COIII, and NAD4, the stop codons of these three PCGs are incomplete TA* (NAD1, NAD4) and T** (COIII). For these incomplete stop codons, we speculate that the loss of nucleotides may be due to polyadenylation of DNA during transcription, which is normal in vertebrate mitosis.

### 4.2. Phylogenetic Analyses

The mitochondrial sequences have been widely used to infer phylogenetic relationships between bird species [1]. Based on 13 PCGs, the phylogeny of *M. caeruleus* was studied in this study. The results show that *Saxicola* is sister to *Monticola*, and together these are sister to *Oenanthe* (BI bootstrap support 0.97), which is similar to other studies [36]. The grouping of *Saxicola*, *Monticola*, *Oenanthe*, and *Phoenicurus* as sisters to Muscicapa supports the idea of Fengjun Li and Min Zhao et al. [11,35]. Moreover, in the phylogenetic trees, the Muscicapa was a deep branch, which differs from the results of other studies [24].

The relationships of the large branches to which *M. caeruleus* belongs are: ((((*Monticola* + *Saxicola*) + *Oenanthe*) + *Phoenicurus*) + *Myophonus*).

## 5. Conclusions

The mitochondrial genome structure of *M. caeruleus* is similar to that of other passerine birds, including three PCGs, two rRNA genes, twenty-two tRNA genes, and a D-loop. In addition to trnQ, trnA, trnN, trnC, trnY, trnS2, trnP, trnE, and ND6 distributed on the L chain, the other protein-coding genes were evenly distributed on the H chain. There were 24 mismatches in 22 tRNA secondary structures, all of which were G–U mismatches. There were five types of stop codons. There was a control area between trnE and trnF. In the phylogenetic analysis, the two species (((*Monticola*+ *Saxicola*) + *Oenanthe*) + *Phoenicurus*) were sister to *M. caeruleus*.

## Figures and Tables

**Table 1:** List of the 17 Muscicapidae species and one outgroup used in this paper with their GenBank accession numbers.

Family	Genus	Species	GenBank No.	Reference
Paradoxornithidae	*Paradoxornis*	*Paradoxornis heudei*	NC_046943	Unpublished
Muscicapidae	*Myophonus*	*Myophonus caeruleus*	MN564936	This study
	*Ficedula*	*Ficedula albicilla*	MN125374	[23]
		*Ficedula hyperythra*	NC_058320	[24]
		*Ficedula zanthopygia*	OL639031	[25]
	*Niltava*	*Niltava davidi*	NC_039538	[24]
	*Muscicapa*	*Muscicapa sibirica*	NC_045374	[26]
		*Muscicapa latirostris*	NC_045375	[27]
	*Copsychus*	*Copsychus sechellarum*	MN356447	[24]
	*Cercotrichas*	*Cercotrichas coryphoeus*	MN356422	Unpublished
	*Saxicola*	*Saxicola maurus*	MN356403	Unpublished
	*Monticola*	*Monticola gularis*	NC_033536	[28]
	*Oenanthe*	*Oenanthe oenanthe*	NC_051036	[29]
		*Oenanthe isabellina*	NC_040290	[30]
	*Cyornis*	*Cyornis hainanus/rubeculoides*	HQ896033	Unpublished
	*Phoenicurus*	*Phoenicurus auroreus*	MT366880	[31]
		*Phoenicurus phoenicurus*	MN122900	Unpublished

**Table 2:** Mitochondrial genome characteristics of *M.caeruleus*.

Feature	Strand	Position	Length (bp)	Initiation Codon	Stop Codon	Anticodon
trnF	H	1–68	68			GAA
rrnS	H	68–1047	980			
trnV	H	1047–1116	70			TAC
rrnL	H	1133–2711	1579			
trnL2	H	2714–2788	75			TAA
NAD1	H	2794–3764	971	ATG	TA(A)	
trnI	H	3779–3850	72			GAT
trnQ	L	3855–3925	71			TTG
trnM	H	3925–3993	69			CAT
NAD2	H	3994–5034	1041	ATG	TAA	
trnW	H	5034–5104	71			TCA
trnA	L	5106–5174	69			TGC
trnN	L	5179–5251	73			GTT
trnC	L	5252–5318	67			GCA
trnY	L	5318–5388	71			GTA
cox1	H	5390–6940	1551	GTG	AGG	
trnS2	L	6932–7006	75			TGA
trnD	H	7010–7078	69			GTC
cox2	H	7087–7770	684	ATG	TAA	
trnK	H	7772–7839	68			TTT
atp8	H	7841–8008	168	ATG	TAA	
atp6	H	7999–8682	684	ATG	TAA	
cox3	H	8688–9471	784	ATG	T(AA)	
trnG	H	9472–9540	69			TCC
NAD3	H	9541–9891	351	ATG	TAA	
trnR	H	9893–9962	70			TCG
NAD4l	H	9964–10,260	297	ATG	TAA	
NAD4	H	10,254–11,631	1378	ATG	T(AA)	
trnH	H	11,632–11,702	71			GTG
trnS1	H	11,703–11,768	66			GCT
trnL1	H	11,768–11,838	71			TAG
NAD5	H	11,839–13,656	1818	ATG	AGA	
cob	H	13,665–14,807	1143	ATG	TAA	
trnT	H	14,811–14,879	69			TGT
trnP	L	14,886–14,955	70			TGG
NAD6	L	14,973–15,491	519	ATG	TAG	
trnE	L	15,493–15,564	72			TTC
OH	H	15,569–16,518	950			

**Table 3:** Base composition of the complete mitochondrial genome, protein coding gene, and rRNA gene of *M. caeruleus*.

Region	A%	C%	G%	T%	A + T%	G + C%	AT Skew	GC Skew
Whole genome	30.06	31.45	14.78	23.71	53.77	46.23	0.12	−0.36
rrnS	31.22	26.94	21.12	20.71	51.94	48.06	0.20	−0.12
rrnL	34.39	23.94	20.39	21.28	55.67	44.33	0.24	−0.08
NAD1	26.67	32.75	14.52	26.06	52.73	47.27	0.01	−0.39
NAD2	30.74	34.1	10.85	24.3	55.04	44.96	0.12	−0.52
cox1	27.79	30.5	17.15	24.56	52.35	47.65	0.06	−0.28

**Table 4:** Codon number and relative synonymous codon usage (RSCU) of *M. caeruleus* mitochondrial protein-coding genes (PCGs).

Codon	Count	RSCU	Codon	Count	RSCU
UUU (F)	67	0.69	UCU (S)	96	0.97
UUC (F)	128	1.31	UCC (S)	177	1.78
UUA (L)	83	0.73	UCA(S)	130	1.31
UUG (L)	38	0.34	UCG (S)	40	0.4
CUU (L)	98	0.87	CCU (P)	150	1.19
CUC (L)	128	1.13	CCC (P)	160	1.26
CUA (L)	265	2.35	CCA (P)	154	1.22
CUG (L)	66	0.58	CCG (P)	42	0.33
AUU (I)	83	0.68	ACU (T)	116	0.91
AUC (I)	176	1.44	ACC (T)	196	1.54
AUA (I)	107	0.88	ACA (T)	155	1.22
AUG (M)	62	1	ACG (T)	43	0.34
GUU (V)	37	0.91	GCU (A)	65	0.78
GUC (V)	48	1.19	GCC (A)	153	1.84
GUA (V)	60	1.48	GCA (A)	94	1.13
GUG (V)	17	0.42	GCG (A)	21	0.25
UAU (Y)	78	0.77	UGU (C)	32	0.79
UAC (Y)	125	1.23	UGC (C)	49	1.21
UAA (*)	122	1.28	UGA (*)	77	0.81
UAG (*)	86	0.91	UGG (W)	33	1
CAU (H)	89	0.88	CGU (R)	34	0.78
CAC (H)	114	1.12	CGC (R)	33	0.76
CAA (Q)	162	1.47	CGA (R)	44	1.01
CAG (Q)	59	0.53	CGG (R)	28	0.64
AAU (N)	84	0.68	AGU (S)	45	0.45
AAC (N)	162	1.32	AGC (S)	107	1.08
AAA (K)	149	1.35	AGA (R)	66	1.51
AAG (K)	72	0.65	AGG (R)	57	1.31
GAU (D)	50	0.85	GGU (G)	38	0.81
GAC (D)	68	1.15	GGC (G)	47	1.01
GAA (E)	86	1.25	GGA (G)	63	1.35
GAG (E)	52	0.75	GGG (G)	39	0.83

Note: * stands for termination codon.

## Data Availability

The original data presented in the study are openly available in GenBank at accession number MN564936.
